# Deciphering the Molecular Signature of Human Hyalocytes in Relation to Other Innate Immune Cell Populations

**DOI:** 10.1167/iovs.63.3.9

**Published:** 2022-03-10

**Authors:** Julian Wolf, Stefaniya Boneva, Dennis-Dominik Rosmus, Hansjürgen Agostini, Günther Schlunck, Peter Wieghofer, Anja Schlecht, Clemens Lange

**Affiliations:** 1 Eye Center, Medical Center, Faculty of Medicine, University of Freiburg, Baden-Wuerttemberg, Germany; 2 Institute of Anatomy, University of Leipzig, Leipzig, Saxony, Germany; 3 Cellular Neuroanatomy, Institute of Theoretical Medicine, Medical Faculty, University of Augsburg, Bavaria, Germany; 4 Institute of Anatomy and Cell Biology, Julius-Maximilians-University Wuerzburg, Wuerzburg, Bavaria, Germany; 5 Ophtha-Lab, Department of Ophthalmology, St. Franziskus Hospital, Muenster, Germany

**Keywords:** hyalocytes, vitreous body macrophages, retinal microglia, monocytes, ocular immune privilege, RNA sequencing, transcriptional profile

## Abstract

**Purpose:**

Hyalocytes are the tissue-resident innate immune cell population of the vitreous body with important functions in health and vitreoretinal disease. The purpose of this study is to gain new insights into the biology and function of human hyalocytes in comparison to other innate immune cells.

**Methods:**

The present study applies fluorescence-activated cell sorting and RNA sequencing to compare the transcriptional profiles of human hyalocytes, retinal microglia (rMG) and classical, intermediate, and non-classical monocytes isolated from the same patients. Immunohistochemistry was applied for morphological characterization of human hyalocytes.

**Results:**

Pairwise analysis indicates distinct differences between hyalocytes and monocytes, whereas a high degree of similarity to rMG is apparent, with comparable expression levels of established microglia markers, such as *TREM2*, *P2RY12*, and *TMEM119*. Among the top expressed genes in hyalocytes, *SPP1*, *CD74*, and *C3*, were significantly upregulated when compared with monocytes. Despite the high level of similarity of hyalocytes and rMG, ten highly expressed genes in hyalocytes compared to microglia were identified, among them *FOS*, *DUSP1*, and *EGR2*.

**Conclusions:**

This study reveals a high degree of similarity between hyalocytes and retinal microglia. Nevertheless, hyalocytes exhibit some expression differences that may adapt them to the specific needs of the vitreous and provide the basis for deciphering the multiple roles of this fascinating cell population in health and vitreoretinal diseases.

Hyalocytes represent the tissue-resident macrophages of the vitreous body with important functions in health and vitreoretinal disease.^1^^–^^4^ Although hyalocytes were first described in 1840,^5^ were suggested to be of leukocyte origin 3 decades later,^6^ and their structural and ultrastructural characteristics were described in detail by Sebag in 1989,^7^ until now, little has been known about the biology, function, and relationship of human hyalocytes in comparison with other ocular myeloid cells. Functionally, hyalocytes were described to be involved in the synthesis of vitreal extracellular matrix proteins, such as hyaluronan, glycoproteins, and collagen,^1^ and to play a role in inhibiting intraocular inflammation as part of the ocular immune privilege.^1^^,^^3^ Thus, they were suggested to provide protection against immune-mediated inflammatory damage to maintain a clear optical axis and optimal visual acuity.^1^^,^^3^ At the same time, hyalocytes are regarded as important modulators of intravitreal inflammation, suggesting a role in pathological conditions of the vitreoretinal interface, such as proliferative vitreoretinopathy,^8^ macular pucker,^8^ or proliferative diabetic retinopathy (PDR).^1^^,^^9^ We have previously established a protocol for isolation of human hyalocytes using fluorescence-activated cell sorting (FACS) and to characterize their transcriptional profile using RNA sequencing.^3^ However, until now, the function and relationship of hyalocytes to other ocular myeloid cells, such as retinal microglia or infiltrating monocytes, remain unclear or have been investigated in other species than humans with outdated methodology.^10^

The present study describes the FACS-isolation of human hyalocytes, as well as retinal microglia, and classical, intermediate, and non-classical monocytes from nine patients. This study design enables the comparison of human hyalocytes with retinal microglia as well as infiltrating monocytes, which was previously unknown, and at the same time minimizes technical and interindividual variation. The results provide detailed insights into the transcriptional profile of human hyalocytes, indicating a high level of similarity to retinal microglial cells. In addition, this study identifies overexpressed genes in hyalocytes as well as several inflammatory and immunomodulatory factors expressed in hyalocytes that may form the basis for understanding hyalocytes as key regulatory cells of vitreal homeostasis and vitreoretinal disease.

## Methods

### Patients

A total of six patients undergoing enucleation due to melanoma of the iris, ciliary body, or choroid at the University Eye Hospital Freiburg between 2018 and 2020 were included in this study. Diagnosis was made based on anterior segment and fundus examination and photography, optical coherence tomography, ultrasound, and ultrasound biomicroscopy (Supplementary Fig. S1). Immediately after enucleation, eyes were dissected in the operating theater. In a first step, vitreous was aspirated through a small incision in a region not affected by the tumor. Subsequently, the eye was divided into two parts, one containing the tumor and one with healthy tissue. The part containing the tumor was referred to routine ophthalmopathologic examination, whereas the healthy part was used to collect retinal tissue from the macular region. Vitreous and retinal tissue were placed on ice immediately after dissection and processed within two hours after surgery for isolation of hyalocytes or retinal microglia, respectively. In addition, whole blood was obtained from three of the patients undergoing enucleation as well as from three age- and sex-matched individuals and processed within two hours for isolation of classical, intermediate, and non-classical monocytes. The study protocol was reviewed and approved by the local ethics committee (University Freiburg, Germany, approval number 20-1165). Written informed consent was obtained from each patient.

### Fluorescence-Activated Cell Sorting

Hyalocytes were isolated from vitreous samples, retinal microglia from retinal tissue, and classical, intermediate, and non-classical monocytes from whole blood using FACS, as previously described.^3^ To generate a single cell suspension, vitreous samples were digested with Collagenase D (5 mg/mL, 2.5 mg in 0.5 mL vitreous) and DNase I (1 mg/mL) in Hanks balanced salt solution (HBSS) for 20 minutes at 37°C. Retinal tissue was dissociated by resuspension using pipettes. After filtering cell solutions through a 70 µm cell strainer, 0.5 µL of Fixable Viability Dye (eFluor 780; eBioscience) per 1 mL of cell solution was added. In the following, anti-CD16/CD32 Fc block (BD Pharmingen) was performed at 4°C for 20 minutes at a concentration of 1:200 to avoid unspecific binding. Retinal and vitreal cells were stained for CD45 (BV421, anti-human, 1:100; BioLegend), CD11b (FITC, anti-human, 1:100; BioLegend), CX_3_CR1 (PE-Cy7, anti-human, 1:200; BioLegend), and CCR2 (PerCP/Cy5.5, anti-human, 1:200; BioLegend). To exclude any potential contamination with blood-derived monocytes due to possible surgically induced micro bleedings, we further used the Anti-Human Mature Macrophages (MatMac) antibody, an ED2-like (ectodermal dysplasia 2) marker for resident macrophages, which is absent in blood-derived monocytes^11^^–^^14^ (eFluor660, anti-human, 1:100; eBioscience). For isolation of monocytes, peripheral blood mononuclear cells (PBMCs) were separated from the buffy coat using density gradient centrifugation (Ficoll-Hypaque; Anprotec) according to the manufacturer's instructions. PBMCs were stained for CD45 (BV421, anti-human, 1:100; BioLegend), CD11b (FITC, anti-human, 1:100; BioLegend), CX_3_CR1 (PE-Cy7, anti-human, 1:200; BioLegend), CCR2 (APC/Cy7, anti-human, 1:200; Biolegend), CD14 (PE, anti-human, 1:200; BioLegend), and CD16 (BV711, anti-human, 1:200; BioLegend). After an incubation step of 20 minutes at 4°C, cells were resuspended in FACS buffer and processed for sorting on the FACS Fusion (BD Pharmingen). Viable single cells (doublet exclusion) were sorted into RNA Protect Cell Reagent (QIAGEN) at 2 to 8°C until sequencing was performed, as previously described.^3^

### Total RNA Extraction

Total RNA was extracted from FACS sorted cells stabilized in RNAprotect buffer according to the “Purification of total RNA from animal and human cells” protocol of the RNeasy Plus Micro Kit (QIAGEN, Hilden, Germany). In brief, cells were stored and shipped in RNAprotect buffer at 2 to 8°C. After pelleting by centrifugation for 5 minutes at 5000 × g, RNAprotect was replaced by 350 µL RLT Plus buffer and the samples were homogenized by vortexing for 30 seconds. Genomic DNA contamination was removed by using gDNA Eliminator spin columns. Next, one volume of 70% ethanol was added and the samples were applied to RNeasy MinElute spin columns followed by several wash steps. Finally, the total RNA was eluted in 12 µL of nuclease-free water. Purity and integrity of the RNA were assessed on the Agilent 2100 Bioanalyzer with the RNA 6000 Pico LabChip reagent set (Agilent, Palo Alto, CA, USA).

### RNA-Sequencing

The SMARTer Ultra Low Input RNA Kit for Sequencing version 4 (Clontech Laboratories, Inc., Mountain View, CA, USA) was used to generate first strand cDNA from approximately 1 ng total-RNA. Double-stranded cDNA was amplified by LD PCR (12 cycles) and purified via magnetic bead clean-up. Library preparation was carried out as described in the Illumina Nextera XT Sample Preparation Guide (Illumina, Inc., San Diego, CA, USA). Then, 150 pg of input cDNA were tagmented (tagged and fragmented) by the Nextera XT transposome. The products were purified and amplified via a limited-cycle PCR program to generate multiplexed sequencing libraries. For the PCR step, 1:5 dilutions of index 1 (i7) and index 2 (i5) primers were used. The libraries were quantified using the KAPA Library Quantification Kit - Illumina/ABI Prism User Guide (Roche Sequencing Solutions, Inc., Pleasanton, CA, USA). Equimolar amounts of each library were sequenced on a NextSeq 500 instrument controlled by the NextSeq Control Software (NCS) version 2.2.0, using two 75 Cycles High Output Kits with the dual index, single-read (SR) run parameters. Image analysis and base calling were done by the Real Time Analysis Software (RTA) version 2.4.11. The resulting .bcl files were converted into .fastq files with the bcl2fastq version 2.18 software. RNA extraction, library preparation, and RNAseq were performed at the Genomics Core Facility “KFB - Center of Excellence for Fluorescent Bioanalytics” (University of Regensburg, Regensburg, Germany; www.kfb-regensburg.de).

### Bioinformatics

Sequencing data (fastq-files) were uploaded to the Galaxy web platform (usegalaxy.eu),^15^ as previously described.^16^ Quality control was performed with FastQC (Galaxy version 0.72, http://www.bioinformatics.babraham.ac.uk/projects/fastqc/ last access on 03/01/2021). Reads were mapped to the human reference genome (Gencode, GRCh38.p13, all regions, release 37) with RNA STAR (Galaxy version 2.7.7a)^17^ using the Gencode main annotation file (Gencode, release 37). Two BAM files for each sample (one for each lane) were combined in one BAM file per sample using Merge BAM Files (Galaxy version 1.2.0). Reads mapped to the human reference genome were counted with featureCounts (Galaxy version 2.0.1)^18^ using the aforementioned annotation file. The outputs of featureCounts were imported to RStudio (version 1.4.1103, R version 4.0.3). Gene symbols were determined based on the ENSEMBL database (release 103, download on March 23, 2021).^19^ Genes with zero reads in all samples were removed from the analysis. To identify the top expressed genes in hyalocytes, transcripts per million were calculated based on the output of featureCounts (assigned reads and feature length), as previously described.^20^ Expression profiles of all five cell types were adjusted for individual patients by the limma function removeBatchEffect^21^ and by consideration within the linear model of DESeq2.^22^ After principle component analysis,^22^ normalized reads and differential gene expression were calculated using the R package DESeq2 (version 1.30.1) with default parameters (Benjamini-Hochberg adjusted *P* values).^22^ Transcripts with log2 fold change (log2FC) >2 or <-2 and adjusted *P* value < 0.05 were considered as differentially expressed genes (DEG). Heatmaps were created with the R package ComplexHeatmap (version 2.6.2).^23^ Gene ontology analysis and its visualization with dotplots and cnetplots was performed using the R package clusterProfiler (version 3.18.1).^24^ Other data visualization was done using the ggplot2 package (version 3.3.3).^25^ To investigate specificity of expression profiles, known cell type-specific marker genes for different immune cell populations were examined.^26^^,^^27^ In addition, expression levels of the most specific marker genes, known from single-cell RNA sequencing of human retinal tissue,^27^ of potentially contaminating cells, such as photoreceptors, retinal ganglion cells, bipolar cells, and endothelial cells, were studied. To analyze the similarity between transcriptional profiles of hyalocytes and the four other immune cell populations, the expression of each gene in each cell population was calculated as a percentile. The similarity was defined as one – delta percentile and quantified by Pearson correlation coefficient R^2^ (with higher values meaning higher similarity between the 2 compared cell populations). To identify overexpressed genes in hyalocytes, upregulated genes in hyalocytes compared to all other cell populations were determined in a first step (log2FC > 2 and *P* adjusted < 0.05). In a second step, the 10th and 90th percentile of expression for each gene were calculated for each cell type and only genes, where the 10th percentile of expression in hyalocytes was higher than the 90th percentile of expression in all other cell types were retained. The top ten overexpressed genes in hyalocytes were selected according to the specificity for hyalocytes, which was defined as the difference between percentiles. To validate these markers, their expression was analyzed in four independent and publicly available data sets. For this purpose, the transcriptional profiles of hyalocytes were included, which were recently FACS-isolated from patients undergoing vitrectomy due to macular pucker or macular hole by our group^3^^,^^28^ (GEO accession: GSE147657). In addition, the expression profiles of human brain microglia^26^ (GEO accession: GSE99074, samples with origin Netherlands), human monocytes and monocyte-derived macrophages^29^ (GEO accession: GSE58310) were included. To ensure comparability of expression between different samples, all validation and training samples were integrated into one DESeq2 model. Clustering of all samples based on the expression of the top ten overexpressed genes in hyalocytes was performed using t-distributed Stochastic Neighbor Embedding (t-SNE) plots (perplexity = 16, theta = 0.0).^30^

### Immunohistochemistry

For morphologic characterization of human hyalocytes, we obtained the vitreous body samples from a donor eye of a patient (woman aged 82 years) undergoing enucleation at the University Eye Hospital Freiburg in December 2021 due to ciliary body melanoma. Written informed consent was obtained from the patient. Immediately after enucleation, vitreal samples, including vitreal cortex, were obtained by dissection, transferred to a 24-well plate, and incubated in cell culture medium for one night (DMEM, 10% fetal bovine serum, Penicillin 100 U/mL, Streptomycin 100 µg/mL). The next day, the tissue was fixed in 4% paraformaldehyde (PFA) for 20 minutes at 4°C. After washing with phosphate buffered saline (PBS) at room temperature (RT), the tissue was blocked with PBS containing 5% normal goat serum (NGS) and 0.1% Triton X-100 for 3 hours at RT. Next, the tissue was incubated with a primary antibody against AIF1 (IBA1; dilution: 1:500, ab5076; Abcam, Cambridge, United Kingdom) in antibody buffer (PBS containing 1% NGS and 0.1% Triton X-100) overnight at 4°C. The next day, following washing with PBS at RT, the tissue was incubated with a secondary antibody (Alexa 647 donkey anti-goat, 1:500, [A21447]; Life Technologies, USA) and Phalloidin (TRITC-labeled, 1:500, P1951; Sigma-Aldrich, USA) in antibody buffer for 1 hour at RT in the dark. After washing with PBS, nuclei were stained with 4′,6-Diamidin-2-phenylindol (DAPI) 1:1000 in PBS for 10 minutes at RT in the dark and washed again with PBS. To mount the vitreous specimen, a flat mold was prepared on a slide using heated paraffin and a cotton swab to form a frame which held the specimen in Fluorescence Mounting Medium (Agilent Dako, S3023; Dako, USA). The paraffin frame was covered with a coverslip and sealed at the edges with a thin layer of paraffin. Images were taken using a Leica SP8 confocal microscope (Leica, Wetzlar, Germany).

## Results

### Patient's Characteristics

A total of ten patients with a mean age of 70.2 ± 15.2 years, including four women and six men were enrolled in this study. Enucleation was performed in six patients at the University Eye Hospital Freiburg between 2018 and 2020 due to melanoma of the iris (*n* = 1), ciliary body (*n* = 2), or choroid (*n* = 3). Vitreous and retinal tissue were collected from the enucleated eyes and, in addition, whole blood was taken from three of these patients (patients #1, 2, and 5) as well as from three age- and sex-matched individuals (patients #7, 8, and 9). For immunohistochemistry, the vitreous body was obtained from one donor eye undergoing enucleation due to ciliary body melanoma at our institution in December 2021. For all patients, details regarding the diagnosis, including clinical images, are summarized in Supplementary Figure S1. Hyalocytes (CD45^+^, CD11b^+^, CX_3_CR1^+^, MatMac^+^, and CCR2^−^), retinal microglia (CD45^+^, CD11b^+^, CX_3_CR1^+^, MatMac^+^, and CCR2^−^), as well as classical (CD45^+^, CD11b^+^, CX_3_CR1^+^, CD14^++^, and CD16^−^), intermediate (CD45^+^, CD11b^+^, CX_3_CR1^+^, CD14^++^, and CD16^+^), and non-classical monocytes (CD45^+^, CD11b^+^, CX_3_CR1^+^, CD14^−^, and CD16^++^) were FACS-isolated from these tissues (Supplementary Fig. S2). No vitreous was isolated from one patient (patient #3) due to a large choroidal melanoma and one microglia sample (patient #2) had to be excluded due to significant monocyte contamination, as evidenced by high expression levels of monocyte and low expression levels of microglia marker genes.

### Quality of Expression Profiles

Comparing the transcriptional profiles of any 2 patients yielded mean Pearson correlation coefficients of 0.89 (minimum = 0.79 and maximum = 0.95) for hyalocytes, 0.92 (minimum = 0.83 and maximum = 0.96) for retinal microglia, 0.97 (minimum = 0.91 and maximum = 0.99) for classical monocytes, 0.99 (minimum = 0.98 and maximum = 0.99) for intermediate monocytes, and 0.88 (minimum = 0.68 and maximum = 0.98) for non-classical monocytes, indicating high similarities of expression profiles between different patients for all 5 immune cell populations (Supplementary Fig. S3A). In addition, based on known cell type-specific marker genes,^26^^,^^27^^,^^31^^,^^32^ a high specificity of expression profiles was confirmed for all five cell types (see Supplementary Fig. S3B). At the same time, low to absent expression levels of photoreceptor-, retinal ganglion cell-, bipolar cell-, and endothelial cell-specific genes were detected, confirming a low proportion of contaminating cells (see Supplementary Fig. S3B). To evaluate a potential impact of melanoma on the expression profiles, we compared the transcriptional profiles of hyalocytes isolated from enucleated eyes with those of hyalocytes collected from patients undergoing vitrectomy due to macular pucker or macular hole in a previous study by our group.^3^ This analysis revealed high similarity between both groups, as indicated by high Pearson correlation coefficients of any two patients in both groups (mean = 0.89, minimum = 0.68, and maximum = 0.98) and the absence of a group-specific clustering in an unsupervised t-Distributed Stochastic Neighbor Embedding cluster analysis (Supplementary Figs. S4A and S4B). These results indicate that a significant melanoma effect on the expression profiles is unlikely.

### Transcriptional Characterization of Human Hyalocytes

**Figure 1.**
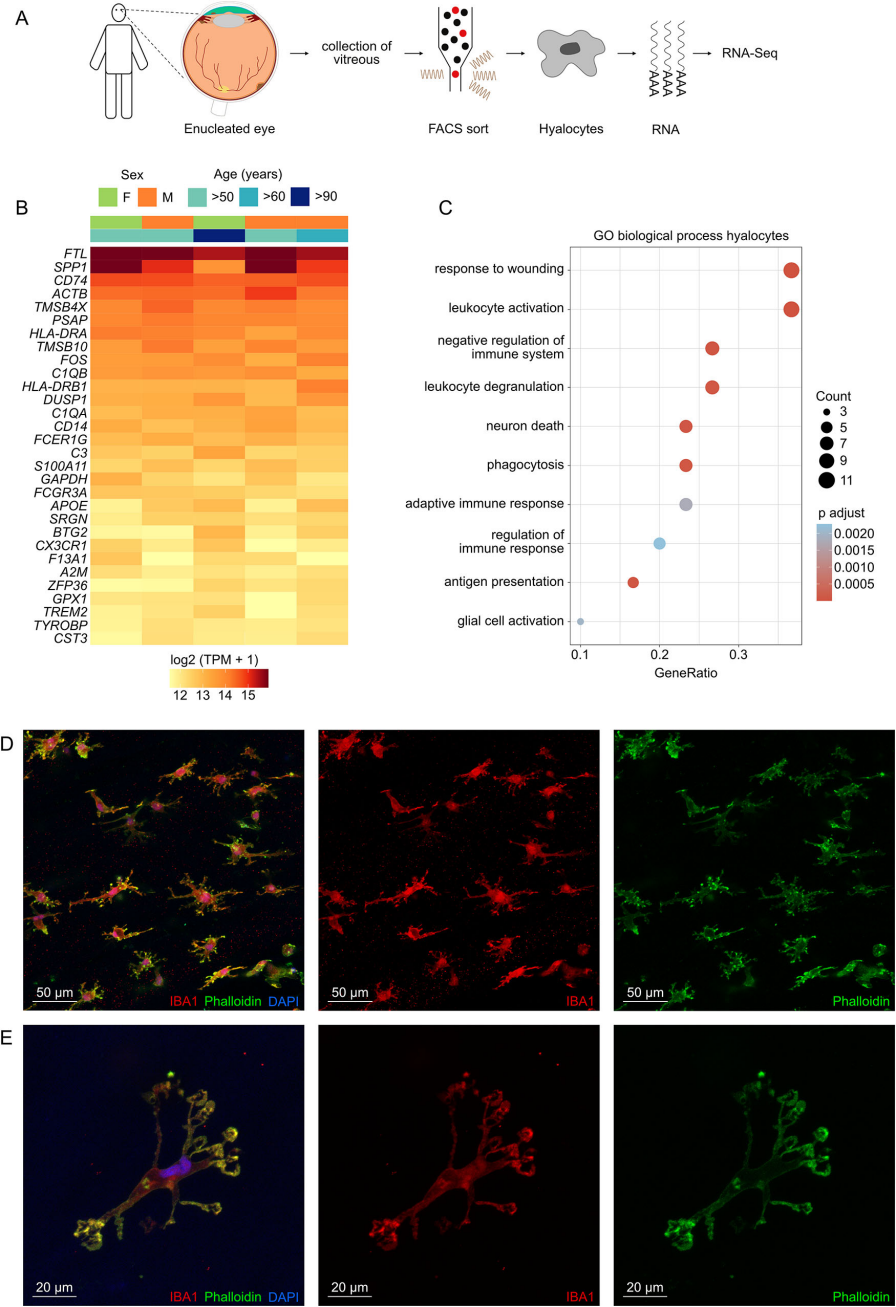
Transcriptional characterization of human hyalocytes. (**A**) Experimental setup: vitreous was collected from five eyes enucleated due to melanoma of the iris, ciliary body or choroid and hyalocytes (CD45^+^, CD11b^+^, CX_3_CR1^+^, MatMac^+^, and CCR2^−^) were isolated by Fluorescence-Activated Cell Sorting (FACS) followed by RNA sequencing. (**B**) Heatmap of the top 30 genes expressed in hyalocytes ordered by mean expression. Each column represents one sample and each row one gene. TPM, transcripts per million. (**C**) Dotplot visualizing the top 10 gene ontology (GO) biological processes, which the top 30 genes expressed in hyalocytes were involved in. The size of the dots represents the number of associated genes (count). The adjusted *P* value of each GO term is shown by color. (**D,** **E**) Morphologic characterization of human hyalocytes obtained from the vitreous of a donor eye (enucleated due to ciliary body melanoma) using immunohistochemical staining against IBA1 (*red*), phalloidin (*green*), and DAPI (*blue*), including an overview of several hyalocytes (**D**) and a more detailed view of one hyalocyte (**E**).

Hyalocytes were FACS-isolated from the vitreous of five patients undergoing enucleation due to melanoma of the iris, ciliary body or choroid (Fig. 1A). Transcriptome analysis revealed that in addition to numerous leukocyte markers, such as *PTPRC* (CD45), *ITGAL* (CD11a), and *ITGAM* (CD11b), human hyalocytes expressed high levels of several microglia markers, such as *TREM2* (triggering receptor expressed on myeloid cells 2), *P2RY12* (purinergic receptor P2Y12), and *TMEM119* (transmembrane protein 119; see Supplementary Fig. S3B). The expression profile of the top expressed genes in human hyalocytes is visualized in Figure 1B, indicating no significant age- or sex-dependent variations. The top five expressed genes were *FTL* (ferritin light chain), *SPP1* (secreted phosphoprotein 1), *CD74* (CD74 molecule), *ACTB* (actin beta), and *TMSB4X* (thymosin beta 4 X-linked). Gene Ontology (GO) analysis revealed that the top expressed genes in human hyalocytes contributed most significantly to biological processes, such as response to wounding (GO: 0009611, adjusted *P* < 1.3e-6), leukocyte activation (GO: 0002274, adjusted *P* < 1.3e-6), negative regulation of immune system (GO: 0002683, adjusted *P* < 6.5e-5, GO: 0002764, adjusted *P* < 2.3e-3), phagocytosis (GO: 0006909, adjusted *P* < 8.9e-5), adaptive immune response (GO: 0002250, adjusted *P* < 1.9e-3), and antigen presentation (GO: 0002504, adjusted *P* < 6.9e-5; see Fig. 1C). The contribution of each gene in these biological processes is visualized in the network diagram in Supplementary Figure S5, revealing that genes, such as *FCER1G* (Fc fragment of IgE receptor Ig), *TYROBP* (transmembrane immune signaling adaptor TYROBP), *CD74*, *TREM2*, *HLA-DRB1* (major histocompatibility complex, class II, DR beta 1), and *C1QA* (complement C1q A chain) were key contributors to these processes. In addition, several genes involved in vitreous extracellular matrix metabolism, including glycoproteins, such as aggrecan (*ACAN*) and versican (*VCAN*), as well as several collagen types, such as *COL8A2*, *COL9A2*, and *COL5A1*, were found to be strongly expressed in hyalocytes (Supplementary Fig. S6). For morphologic characterization of human hyalocytes, the vitreous of a donor eye (enucleated due to ciliary body melanoma), was transferred to cell culture and subsequently immunohistochemically stained against IBA1, phalloidin, and DAPI (see Figs. 1D, 1E). These analyses revealed a highly branched morphology of human hyalocytes with many elongated cell extensions reminiscent of retinal microglia morphology.

### Transcriptional Profiling of Human Hyalocytes Compared to Retinal Microglia and Monocytes

**Figure 2.**
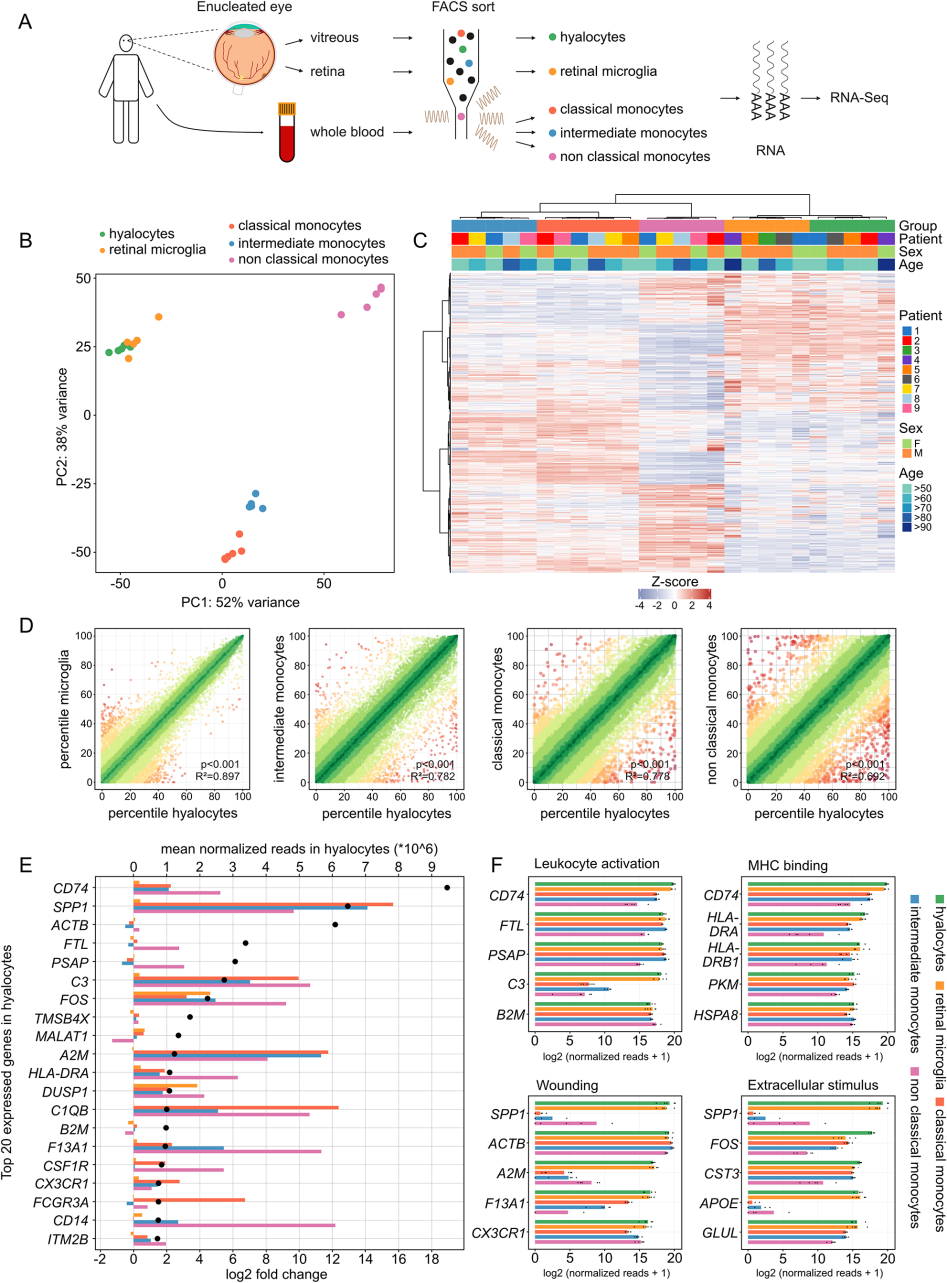
Transcriptional profile of hyalocytes compared to retinal microglia and monocytes. (**A**) Experimental setup: vitreous and retinal tissue from the macular region was collected from six enucleated eyes. In addition, whole blood was collected from six patients. Hyalocytes (CD45^+^, CD11b^+^, CX_3_CR1^+^, MatMac^+^, and CCR2^−^), retinal microglia (CD45^+^, CD11b^+^, CX_3_CR1^+^, MatMac^+^, and CCR2^−^), as well as classical (CD45^+^, CD11b^+^, CX_3_CR1^+^, CD14^++^, and CD16^−^), intermediate (CD45^+^, CD11b^+^, CX_3_CR1^+^, CD14^++^, and CD16^+^), and non-classical monocytes (CD45^+^, CD11b^+^, CX_3_CR1^+^, CD14^−^, and CD16^++^) were isolated by fluorescence-activated cell sorting (FACS) followed by RNA sequencing. (**B**) Principal component analysis (PCA) illustrating the clustering of the five analyzed immune cell populations. (**C**) Unsupervised heatmap visualizing the transcriptional profile of hyalocytes, retinal microglia, as well as classical, intermediate and non-classical monocytes (the top 5000 genes in hyalocytes are shown). Rows and columns are clustered according to similarity of the expression. Cell types and demographics are color-coded in the heatmap annotation at the top (see legend on the right, see **B** for cell types). The z-score represents the deviation from a gene's mean expression in standard deviation units. (**D**) Visualization of the similarity of transcriptional profiles between hyalocytes and the four other immune cell populations. The expression of each gene in each cell population was calculated as a percentile (100 = gene with the highest mean expression and 0 = gene with the lowest mean expression). Colors code for the similarity of expression of each gene between the two compared cell types (defined as 1 = delta percentile, green: high similarity, red = low similarity). The similarity is quantified by Pearson R^2^ (with higher values meaning higher similarity between the two compared cell populations). (**E**) Top 20 highest expressed genes in hyalocytes and their expression in the other four immune cell populations. Genes are ordered according to their mean expression in hyalocytes (mean normalized reads, black dots, upper x-axis). The color bars code for the log2 fold change (lower x-axis) of expression in hyalocytes in comparison to retinal microglia (orange), classical monocytes (*red*), intermediate monocytes (*blue*), and non-classical monocytes (*purple*). A positive log2-fold change corresponds to a higher expression in hyalocytes. (**F**) Bar graphs illustrating the top five expressed genes associated to the most significantly enriched GO biological processes in hyalocytes. The expression is also visualized for the other four analyzed cell types. The lengths of the bars correspond to mean expression, whereas the black dots visualize expression in each sample.

Human hyalocytes, retinal microglia, as well as classical, intermediate, and non-classical monocytes were FACS-isolated followed by RNA sequencing to compare their transcriptional profiles (see Fig. 2A). Unsupervised analysis revealed distinct differences in the expression profile of hyalocytes when compared with all three subtypes of monocytes, with the highest discrepancies seen in comparison to non-classical monocytes (see Figs. 2B, 2C). With reference to retinal microglia, there was a high degree of similarity to hyalocytes, although distinct differences were evident in a relatively small number of genes (see Figs. 2B, 2C). Of note, no significant patient-, age-, or sex-dependent variations were apparent, as demonstrated by sample clustering in the heatmap in Figure 2C. The similarities between hyalocytes and the four other immune cell populations were also quantified by calculating the Pearson correlation coefficient between expression profiles (see Fig. 2D). In accordance with the findings above, the highest similarity was found between hyalocytes and retinal microglia (R^2^ = 0.897, *P* < 0.001), followed by intermediate (R^2^ = 0.782, *P* < 0.001), classical (R^2^ = 0.778, *P* < 0.001), and finally non-classical monocytes (R^2^ = 0.692, *P* < 0.001). Looking at the 20 highest expressed transcripts in hyalocytes, it is evident that many of these genes were significantly upregulated in hyalocytes when compared to monocytes, among them *CD74*, *SPP1*, *C3* (Complement C3), *FOS* (Fos proto-oncogene), *A2M* (alpha-2-macroglobulin), *DUSP1* (dual specificity phosphatase 1), *FCGR3A* (Fc Fragment of IgG Receptor IIIa), and *C1QB* (complement C1q B chain). Some of these genes were also upregulated in comparison to retinal microglia, among them *FOS* and *DUSP1*. In contrast, in addition to *FTL* and *PSAP* (prosaposin), the ubiquitously expressed gene *ACTB* encoding cytoskeleton protein actin beta, were similarly expressed in all five cell populations (see Fig. 2E). Several of these factors were also among the top five expressed genes associated to the most significantly enriched GO biological processes in hyalocytes (see Fig. 2F).

### Pairwise Transcriptional Comparison of Hyalocytes and Retinal Microglia

**Figure 3.**
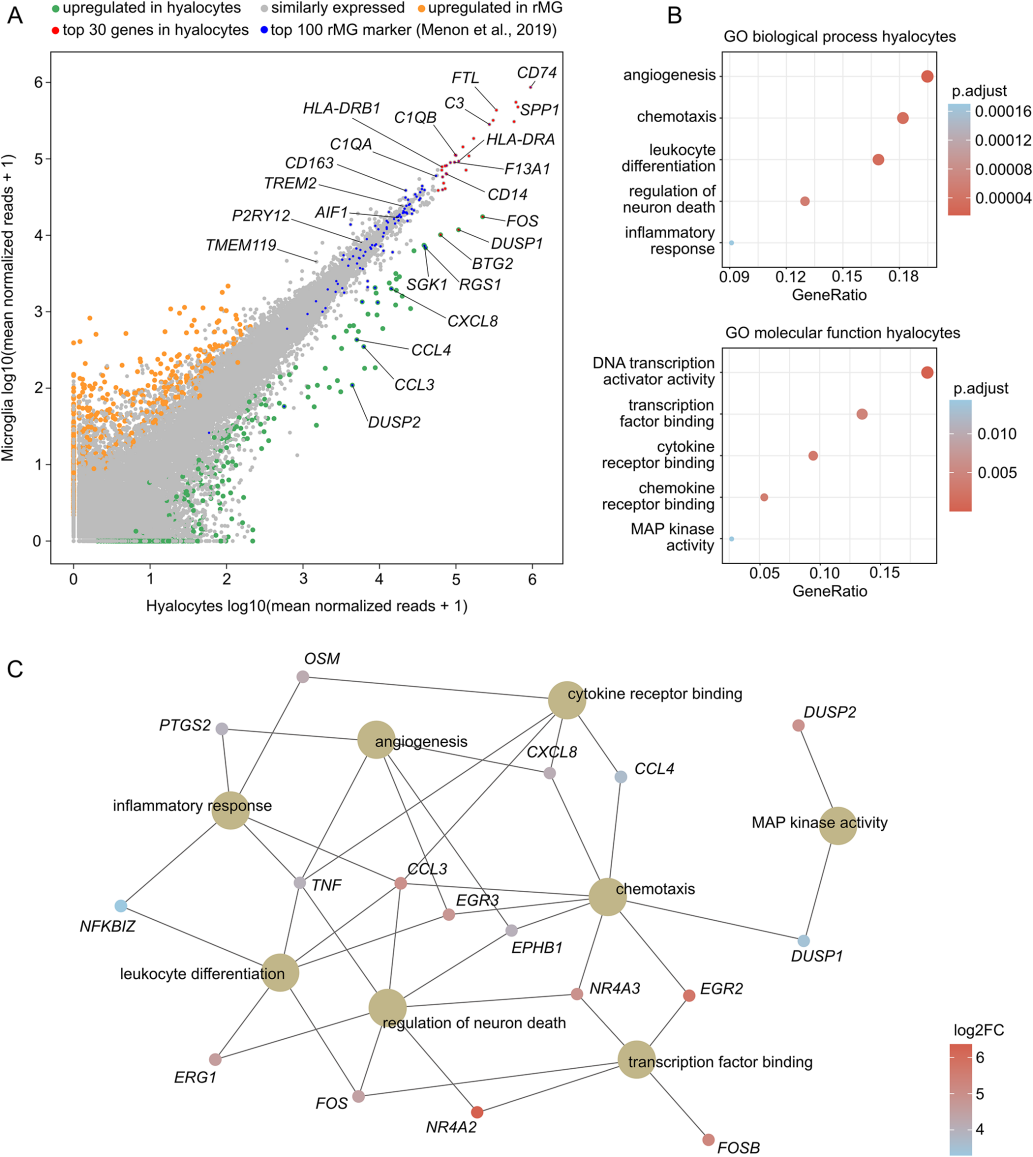
Pairwise transcriptional comparison of hyalocytes and retinal microglia. (**A**) Readplot comparing mean expression of each gene between hyalocytes (x-axis) and retinal microglia (y-axis). Upregulated genes in hyalocytes are colored *green*, and upregulated genes in retinal microglia are shown in *orange*. The top 30 expressed genes in hyalocytes from Figure 1 are marked in red. In addition, the top 100 most specific retinal microglia marker from published single cell RNA-Seq data of the human retina are shown in blue. (**C**) Dotplots visualizing the top five Gene ontology (GO) biological processes and molecular functions, which the upregulated genes in hyalocytes were involved in. The adjusted *P* value of each GO term is shown by color. (**D**) The genes associated with the most significantly enriched GO terms from (**C**) are illustrated in the cnetplot, with the color representing the log2 fold change between hyalocytes and retinal microglia.

Given the high degree of similarity observed between hyalocytes and retinal microglia, the two cell populations were compared in a pairwise analysis in a next step. This comparison revealed that the majority of transcripts (97.8%, *n* = 44289) was expressed at similar levels in both cell populations and that only 1.1% (*n* = 496) and 1.1% (*n* = 508) of genes were found to be up- or downregulated in hyalocytes, respectively (Fig. 3A). Interestingly, most of the top 30 genes in hyalocytes (see Fig. 1B) were expressed at equivalent levels in retinal microglia (see Fig. 3A, red dots). In addition, the majority of 100 microglia-specific marker genes, derived from single-cell RNA sequencing of human retina,^27^ were found to be expressed to a similar extent in hyalocytes (among them *AIF1*, *TREM2*, *P2RY12*, and *TMEM119*; see Fig. 3A, Supplementary Fig. S3B). These results both validate the microglia dataset of this study and further emphasize the high similarity between both cell populations.

**Figure 4.**
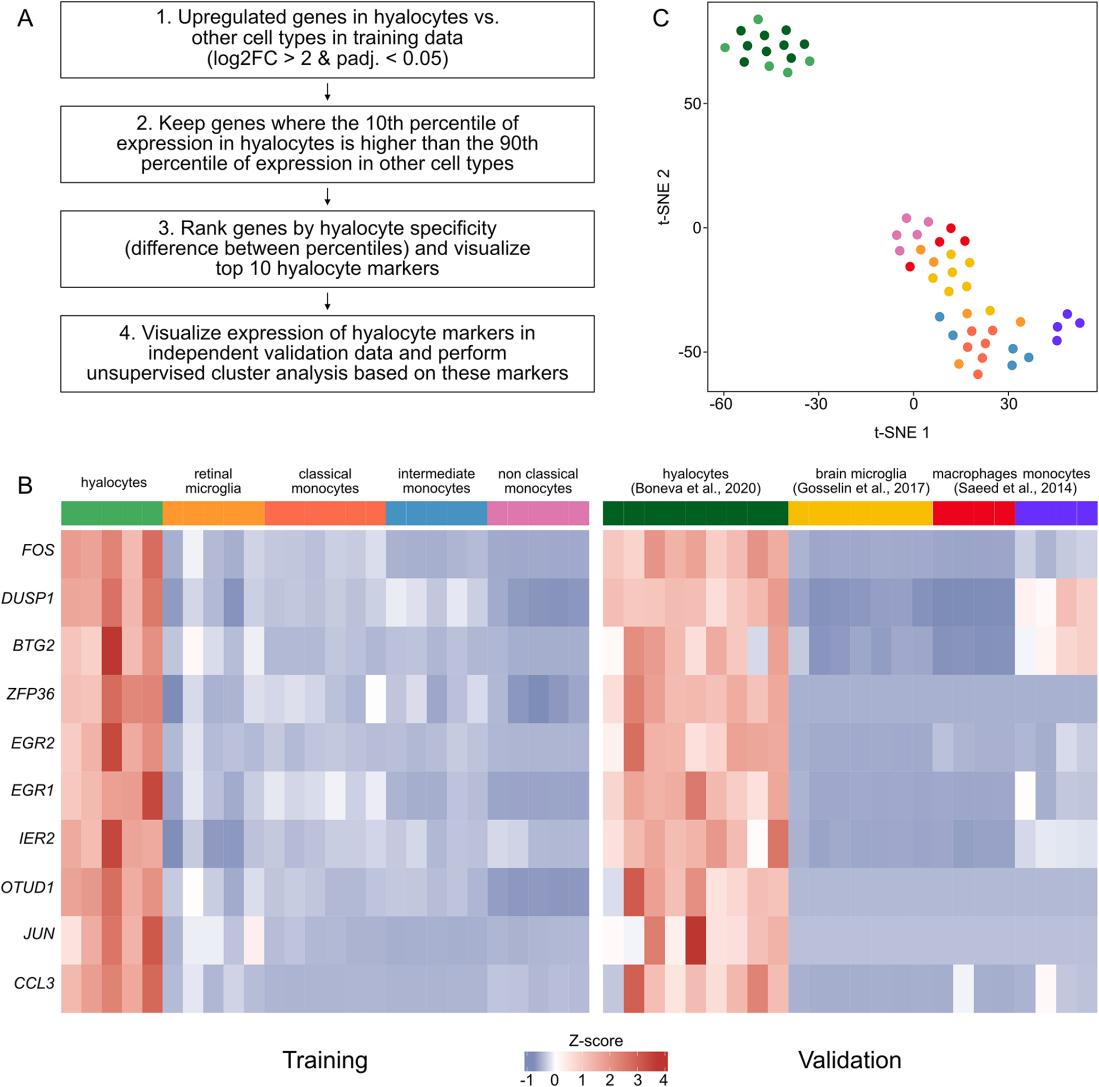
Identification of overexpressed transcripts in hyalocytes. (**A**) Workflow of analysis. (**B**) Heatmap of expression data of the top 10 overexpressed genes in hyalocytes, as identified based on the RNA sequencing data generated in the present study (training data). To validate these markers, expression is also shown in four independent published datasets (validation data). (**C**) Unsupervised cluster analysis using a t-distributed Stochastic Neighbor Embedding (t-SNE) plot based on the 10 marker genes shown in **B** (see color legend in **B**).

Despite the high level of similarity, a total of 496 genes emerged that were significantly higher expressed in hyalocytes than in retinal microglia, with *FOS*, *DUSP1*, and *BTG2* (BTG anti-proliferation factor 2) being among the top differentially expressed genes (see Fig. 3A). The GO analysis revealed that biological processes, such as angiogenesis (GO: 0001525, adjusted *P* < 1.6e-5), chemotaxis (GO: 0006935, adjusted *P* < 3.4e-5), and leukocyte differentiation (GO: 0002521, adjusted *P* < 3.1e-5), as well as molecular functions, such as transcription factor binding (GO: 0008134, adjusted *P* < 1.9e-6), cytokine receptor binding (GO: 0005126, adjusted *P* < 3.1e-3), and MAP kinase activity (GO: 0033549, adjusted *P* < 1.4e-2) were enriched in hyalocytes compared to retinal microglia (see Fig. 3B). The network diagram in Figure 4C illustrates, that genes, such as *TNF* (tumor necrosis factor), *CCL3* (C-C motif chemokine ligand 3), *CXCL8* (C-X-C motif chemokine ligand 8), *EGR3* (early growth response 3), *FOS*, *DUSP1*, and *OSM* (oncostatin M) played key roles in these processes.

### Identification of Overexpressed Transcripts in Hyalocytes

Having identified several overexpressed genes in hyalocytes compared to retinal microglia, we next explored highly expressed transcripts in hyalocytes in comparison to retinal microglia as well as the three subtypes of monocytes. For this purpose, upregulated genes in hyalocytes compared to the four other cell populations were determined and only genes, where the 10th percentile of expression in hyalocytes was higher than the 90th percentile of expression in all other cell types were retained (see Fig. 4A and the Methods section). Genes, such as *FOS*, *DUSP1*, *BTG2*, *ZFP36* (ZFP36 ring finger protein), and *EGR2* (early growth response 2) emerged among the top ten overexpressed genes in hyalocytes (Fig. 4B). To validate these markers, their expression was also analyzed in four independent and publicly available data sets, including human brain microglia,^26^ human monocytes, and monocyte-derived macrophages,^29^ as well as human hyalocytes recently isolated by our group from patients undergoing vitrectomy.^3^ The ten identified markers were also found to be highly expressed in hyalocytes in the validation data (Fig. 4B), which was also evident from the unsupervised cluster analysis based on these 10 factors (Fig. 4C).

## Discussion

Similar to alveolar macrophages in the lung, or Kupffer cells in the liver, hyalocytes represent the tissue-resident macrophages of the vitreous body with important physiologic functions, including synthesis of the extracellular matrix, maintenance of vitreous transparency, and modulation of vitreous immunology.^1^^–^^3^ In addition, hyalocytes are critically involved in the pathophysiology of proliferative diseases of the vitreoretinal interface, such as proliferative vitreoretinopathy,^8^ macular pucker,^8^ or proliferative diabetic retinopathy (PDR).^1^^–^^3^^,^^9^ Although it is widely established that hyalocytes belong to the mononuclear phagocyte system, their relationship and molecular phenotype compared to other ocular and peripheral immune cells, such as retinal microglia or circulating monocytes, is still ill defined. A comparative analysis of these cell populations would be an important prerequisite to understand more precisely the specific role of hyalocytes in comparison to other immune cell populations in healthy individuals and in patients with vitreoretinal diseases. The present study applied FACS and RNA sequencing to compare the transcriptional profiles of human hyalocytes with those of retinal microglia as well as classical, intermediate, and non-classical monocytes providing in-depth insights into the molecular characteristics of all five immune cell populations.

The generated expression profiles revealed high quality and specificity parameters, as indicated by a high similarity between different patients, high cell-specificity based on known cell-specific marker genes, and a low proportion of contaminating cells. Analysis of the transcriptional profile revealed that leukocyte activation, phagocytosis, and antigen presentation were among the most significantly enriched biological processes in hyalocytes, with genes, such as *HLA-DRB1*, *FCER1G*, and *CD74*, identified as central factors in these processes. The analysis further demonstrated that hyalocytes expressed high levels of numerous leukocyte markers, such as *PTPRC* (CD45), *ITGAL* (CD11a), and *ITGAM* (CD11b), confirming the notion that hyalocytes are the tissue-resident macrophages of the vitreous body.^1^^,^^3^^,^^4^^,^^33^ In addition, genes encoding for extracellular matrix glycoproteins, such as aggrecan (*ACAN*) and versican (*VCAN*),^34^ as well as several collagen types, such as *COL8A2*, *COL9A2*, and *COL5A1*, were found to be strongly expressed in hyalocytes, indicating their involvement in vitreous extracellular matrix metabolism. Interestingly, hyalocytes did not express any of the three hyaluronan synthases (*HAS1*-*3*), suggesting that they do not participate in hyaluronan synthesis in the adult eyes. However, they expressed high levels of enzymes involved in hyaluronan degradation (e.g. *HYAL2*), further suggesting a possible role of hyalocytes in modulating vitreous structure.^3^^,^^35^ Additionally, the morphology of human hyalocytes was characterized using immunohistochemical staining against IBA1, phalloidin, and DAPI. These analyses revealed a highly branched morphology of human hyalocytes with many elongated cell extensions, suggesting a high degree of morphological similarity between hyalocytes and retinal microglia.

The comparison of the transcriptional profile of human hyalocytes with those of retinal microglia as well as classical, intermediate, and non-classical monocytes revealed a high degree of similarity between hyalocytes and retinal microglia, whereas distinct differences were apparent when compared to monocytes, most notably non-classical monocytes. These differences were also evident when analyzing the top expressed genes in hyalocytes. Consistent with previous findings,^3^ we found that factors, such as *SPP1*, *CD74*, *C3*, and *C1QB*, were among the top expressed genes in hyalocytes, with these genes being strongly upregulated in hyalocytes as well as in microglia when compared to monocytes. Among them, *SPP1* encoding for Secreted Phosphoprotein 1, emerged as a key factor in vitreous hyalocyte biology. *SPP1*, which encodes for the protein osteopontin, is an extracellular structural protein that plays a central role in the interaction between the innate and adaptive immune system^36^ and is involved in both inflammatory and degenerative processes in the nervous system, as seen in multiple sclerosis and Alzheimer's disease.^37^^–^^39^ In the eye, *SPP1* expressed by retinal microglia was recently identified as a key mediator of retinal inflammation in choroidal neovascularization.^38^ The high level of *SPP1* expression in hyalocytes implies a potential role in inflammatory and neovascular diseases involving the vitreous, such as PDR. *CD74*, which was the highest expressed gene in hyalocytes, has essential roles in antigen presentation by acting as major histocompatibility complex (MHC) class II chaperone, and, in addition, serves as a high affinity receptor for macrophage migration inhibitory factor (MIF).^40^ Soluble CD74 and MIF were found to be increased in the vitreous as well as in endothelial and stromal cells of neovascular membranes in patients with PDR and were shown to have a proangiogenic effect.^41^ Our results indicate that hyalocytes may contribute to the increased CD74 levels in the vitreous of patients with PDR, accounting for an immune modulatory capacity regarding inflammation and angiogenesis in PDR. In addition to these cellular markers of the innate immune system, hyalocytes also expressed components of the complement system, which play an important role in the pathophysiology of diabetic retinopathy^42^ and AMD.^43^ Recent evidence suggests increased levels of complement factor H (CFH) and Complement C3 (C3) in the vitreous of patients with PDR and retinal microglia as a source of CFH^42^ and C3.^27^ Considering our finding of increased C3 expression in hyalocytes, these cells might contribute to the increased C3 levels in the vitreous of patients with PDR, providing further evidence for a possible pathophysiological involvement.

Based on our observation of a high degree of similarity between hyalocytes and retinal microglia, which are important mediators in retinal disease,^44^^–^^46^ we next compared the two cell populations in a pairwise analysis, revealing that 97.8% of transcripts were expressed at similar levels in both cell populations. Interestingly, numerous retinal microglia-specific marker genes, derived from single-cell RNA sequencing,^27^ were also found to be expressed at similar levels in hyalocytes, among them *TREM2*, *P2RY12*, and *TMEM119*. Recently, *TMEM119* was identified as a marker allowing a clear distinction between resident brain microglia and infiltrating myeloid cells due to its high expression in resident brain microglia.^47^

Despite the high level of similarity, pairwise comparison identified 496 genes which were significantly higher expressed in hyalocytes than in retinal microglia. These genes indicated that processes, such as chemotaxis, transcription factor binding, and angiogenesis, were increasingly activated in hyalocytes compared to retinal microglia, with genes, such as *TNF*, *CCL3*, and *CXCL8* playing key roles in these processes. Previous studies have suggested that hyalocytes may participate in pathogenic endothelial cell proliferation by secreting proangiogenic mediators.^48^^,^^49^ TNF alpha plays a central role in PDR as an important proinflammatory cytokine^50^ and, similar to interleukin 8 (encoded by *CXCL8*), was found to be increased in the vitreous in PDR and related to the severity and prognosis of the disease.^51^ CCL3 has also been associated with diabetic retinopathy and the onset of early diabetic retinal damage.^52^ Taken together, these data suggest that hyalocytes may be involved in proinflammatory and proangiogenic processes in diseases, such as PDR, by secreting various proangiogenic and proinflammatory factors into the vitreous.

In search for overexpressed genes in hyalocytes that would in the future allow to differentiate them from other macrophage populations and thus enable the accurate investigation of hyalocyte functions in vitreoretinal disease, this study identified ten marker genes discerning hyalocytes from both retinal microglia and the three subtypes of monocytes. These markers proved to be overexpressed in hyalocytes when tested in four independent and publicly available validation datasets, including human brain microglia,^26^ human monocytes, and monocyte-derived macrophages,^29^ as well as human hyalocytes isolated from patients undergoing vitrectomy due to macular pucker or macular hole.^3^ In addition to *CCL3*, factors such as *FOS* (Fos proto-oncogene, AP-1 transcription factor subunit), *DUSP1* (dual specificity phosphatase 1), *EGR2* (early growth response 2), *BTG2* (BTG anti-proliferation factor 2), and *ZFP36* (ZFP36 ring finger protein) were among these overexpressed genes in hyalocytes. Interestingly, all of these factors have been described as being involved in immunosuppressive processes. As such, the transcription factor *FOS* plays a suppressive role in certain innate and adaptive immune responses including suppression of inflammatory cytokine production and inhibition of T cell activation.^53^^–^^55^ Similarly, *DUSP1* is a negative regulator of innate immune response, as evidenced, for example, by the fact that *DUSP1* is required for the anti-inflammatory effects of glucocorticoids^56^ and is essential in preventing the host from immune-mediated endotoxic shock.^57^ *EGR2* is a transcription factor considered as a negative regulator of the immune response by inhibiting T cell activation^58^ and by anergy induction.^59^ *ZFP36* was shown to restrain T cell activation and expansion.^60^ Finally, *BTG2* was recently identified as a factor responsible for T cell quiescence, an essential process, because inappropriate T cell activation can contribute to the development of various diseases.^61^ In conclusion, these findings reveal that the top five overexpressed genes in hyalocytes are all involved in immunosuppressive processes suggesting a role of hyalocytes in the ocular immune privilege by inhibiting intraocular inflammation.^1^^,^^3^ However, it should be mentioned that genes having immunosuppressive functions in T cells are not necessarily involved in immunosuppressive processes in hyalocytes.

We acknowledge that this study is limited by the use of melanoma eyes, which does not fully exclude the possibility of hyalocytes being activated or affected by the tumor environment. However, the high similarity of the expression profiles of hyalocytes isolated during vitrectomy for macular pucker or macular hole^3^ and those from melanoma eyes argues against a significant melanoma effect. In addition, sample sizes are relatively small, although distinct differences were observed between all five immune cell populations. A strength of this study is the use of fresh tissue, which was processed immediately after enucleation, allowing fixation of sorted cells within four hours after surgery, which is significantly faster compared to other studies using post-mortem tissue with a time from death to preservation of 8 up to 17 hours.^27^^,^^62^ This advantage is of particular relevance considering that retinal tissue can provide stable RNA only if processed within five hours.^63^

In summary, the present study provides detailed insights into the molecular profile of human hyalocytes and indicates a high level of similarity to retinal microglia. Despite the high degree of similarity to retinal microglia, hyalocytes express several distinct factors that identify them as a unique immune cell population adapted to specific needs in the vitreous cavity. As such, hyalocytes express high levels of factors involved in immunosuppressive processes, suggesting a key role in ocular immune privilege. Finally, the identification of several overexpressed genes in hyalocytes may form the basis for deciphering the multiple roles of this fascinating cell population in health and vitreoretinal disease in the future.

## Supplementary Material

Supplement 1
