# Supplementary material for: Deciphering the Molecular Signature of Human Hyalocytes in Relation to Other Innate Immune Cell Populations
Source: Invest Ophthalmol Vis Sci. 2022 Mar 10;63(3):9. doi: 10.1167/iovs.63.3.9 (PMC8934546; doi:10.1167/iovs.63.3.9)
Supplement: Supplement 1 [file iovs-63-3-9_s001.pdf]

Supplement

Deciphering the molecular signature of human hyalocytes in relation to other innate immune cell populations

Julian Wolf[1], Stefaniya Boneva[1], Dennis-Dominik Rosmus[2], Hansjürgen Agostini[1], Günther Schlunck[1], Peter Wieghofer[2,3], Anja Schlecht[1,4 *] and Clemens Lange[1,5*]

[1] Eye Center, Medical Center, Faculty of Medicine, University of Freiburg, Germany

[2] Institute of Anatomy, University of Leipzig, Germany

[3] Cellular Neuroanatomy, Institute of Theoretical Medicine, Medical Faculty, University of Augsburg, Germany

[4] Institute of Anatomy and Cell Biology, Julius-Maximilians-University Wuerzburg, Germany

[5] Ophtha-Lab, Department of Ophthalmology, St. Franziskus Hospital, Muenster, Germany

[*] contributed equally as senior authors

Corresponding author: Clemens Lange MD PhD, Ophtha-Lab, Department of Ophthalmology, St. Franziskus Hospital, Hohenzollernring 74, 48145 Muenster, Germany, tel. +49 251 9352727, fax +49 251 9351111, e-mail: clemens.lange@augen-franziskus.de

Patient 1

Age: 53.4 years Sex: female

Diagnosis: iris melanoma, suspected invasion of iridocorneal angle, secondary glaucoma, iris pigment epithelium intact, vitreous free, fundus without pathological findings

Isolated cells: hyalocytes
retinal microglia
classical monocytes
intermediate monocytes
non classical monocytes

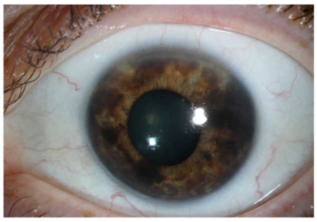

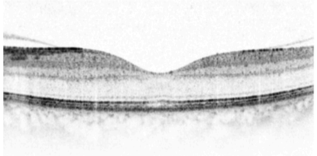

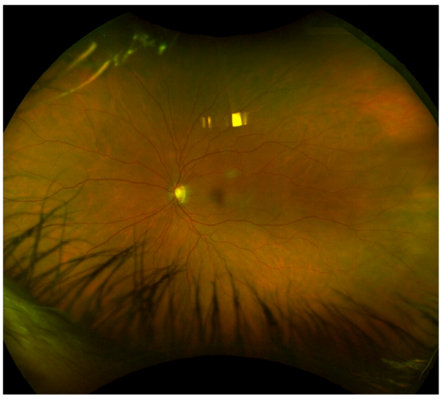

Patient 2

Age: 51.9 years

Sex: male

Diagnosis: choroidal melanoma, collar-button configuration vitreous free, exsudative retinal detachment

Isolated cells: hyalocytes
classical monocytes
intermediate monocytes
non classical monocytes

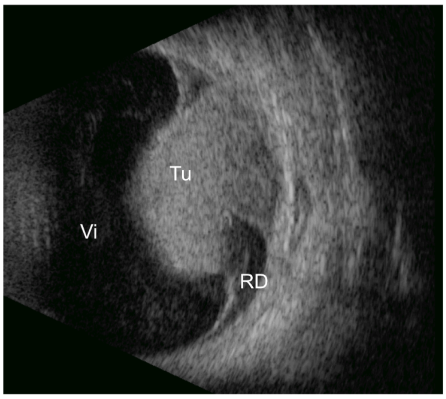

Patient 3

Age: 81.8 years

Sex: male

Diagnosis: choroidal melanoma, large tumor, scleral penetration, no vitreous humor available

Isolated cells: retinal microglia

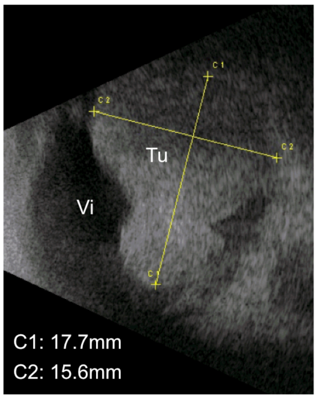

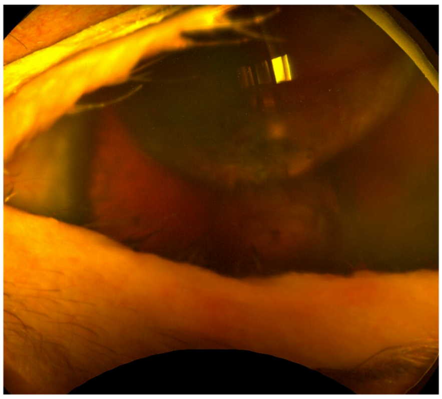

Patient 4

Age: 93.8 years

Sex: female

Diagnosis: ciliary body melanoma, penetration into vortex veins, vitreous and anterior chamber (angle), exsudative retinal detachment

Isolated cells: hyalocytes
retinal microglia

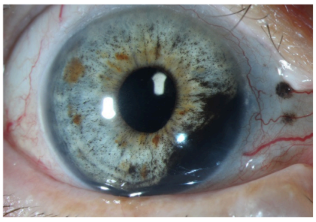

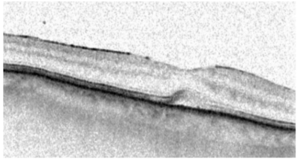

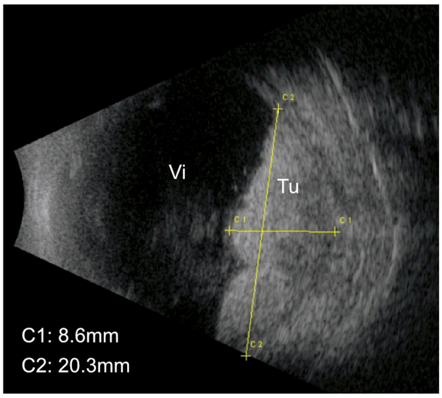

Patient 5

Age: 55.8 years

Sex: male

Diagnosis: choroidal melanoma, monosomie 3, vitreous free, exsudative retinal detachment

Isolated cells: hyalocytes
retinal microglia
classical monocytes

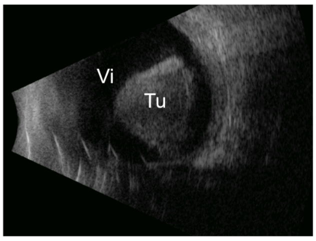

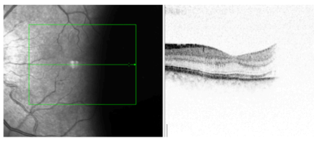

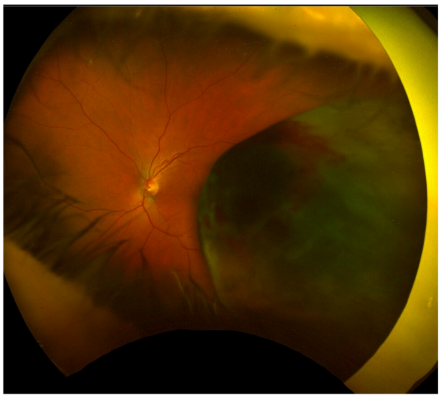

Patient 6

Age: 61.4 years

Sex: male

Diagnosis: ciliary body melanoma, vitreous hemorrhage, exsudative retinal detachment

Isolated cells: hyalocytes
retinal microglia

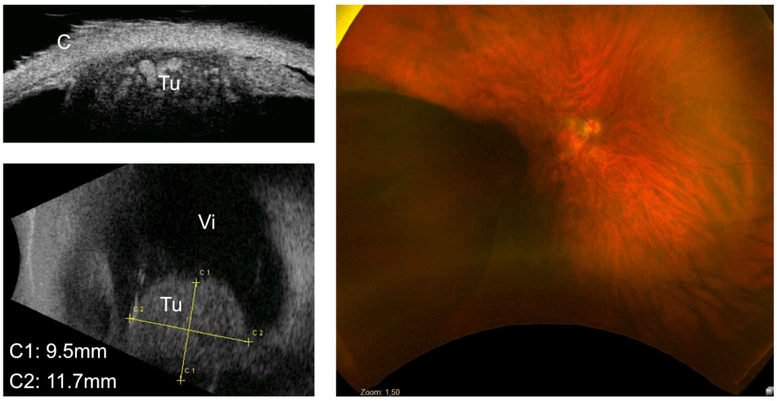

Patient 7

Age: 60.2 years

Sex: male

Diagnosis: naevus of the eyelid

Isolated cells: classical monocytes
intermediate monocytes
non classical monocytes

Patient 8

Age: 84.2 years

Sex: male

Diagnosis: chronic blepharitis

Isolated cells: classical monocytes
intermediate monocytes
non classical monocytes

Patient 9

Age: 77.3 years

Sex: female

Diagnosis: functional nasolacrimal duct obstruction

Isolated cells: classical monocytes
intermediate monocytes
non classical monocytes

Supplementary figure 1: Summary of patients included in the study. Abbreviations: C: cornea, RD: retinal detachment, Tu: tumor, Vi: vitreous.

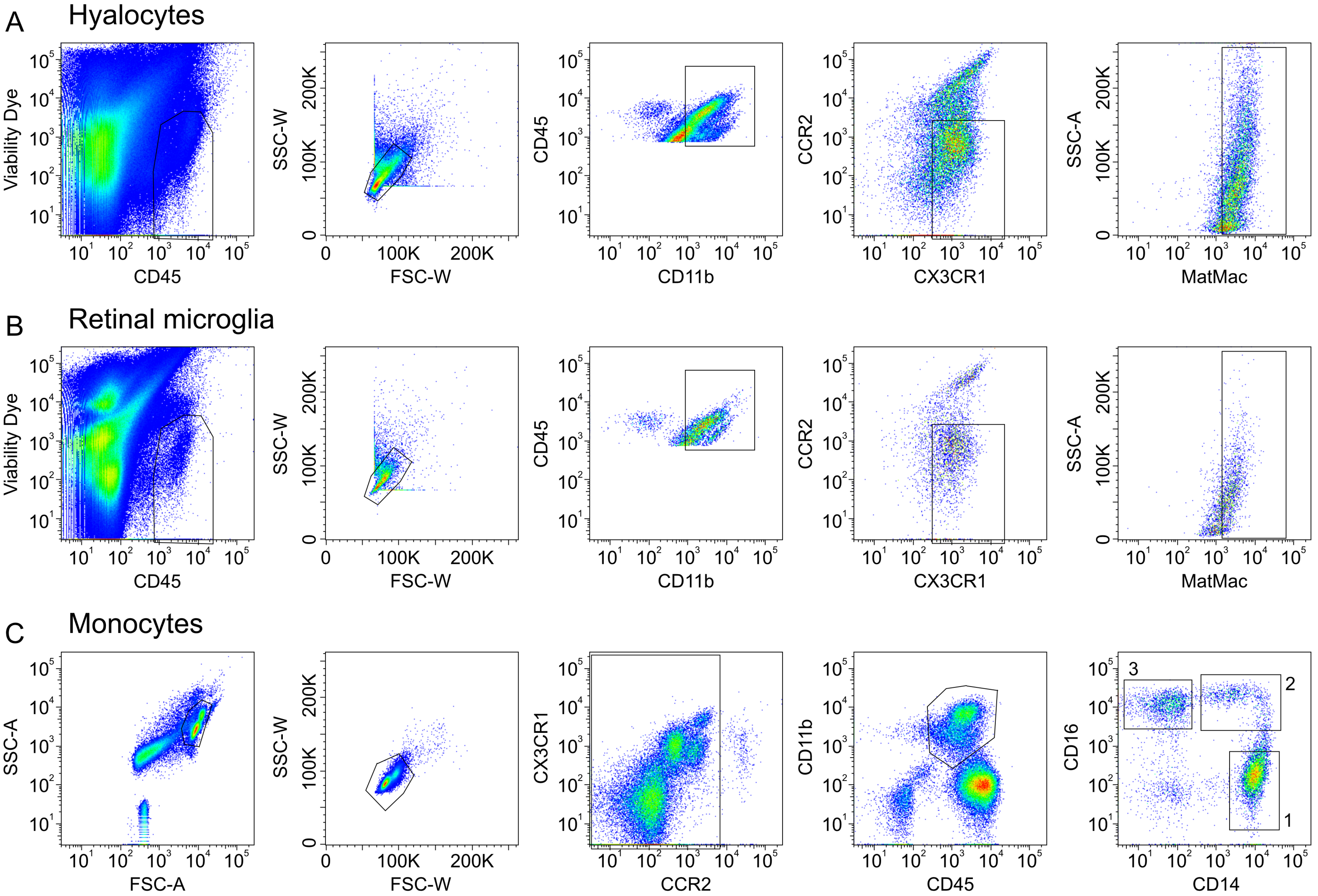

Supplementary figure 2: Flow cytometric gating strategy for (A) human hyalocytes from the vitreous and (B) human retinal microglia from retinal tissue characterized as $CD45^+$ $CD11b^+$ CX_3CR1^+ $CCR2^-$ $MatMac^+$ cells. Cell populations were defined by fluorescence intensity after exclusion of dead cells and doublets by viability dye and physical parameters: FSC: forward scatter, SSC: sideward scatter, W: width, A: Area. (C): In addition, monocytes were isolated from Peripheral Blood Mononuclear Cells obtained from whole blood defined as classical monocytes (1, $CD45^+$ $CD11b^+$ CX_3CR1^+ $CD14^{++}$ $CD16^-$), intermediate monocytes (2, $CD45^+$ $CD11b^+$ CX_3CR1^+ $CD14^{++}$ $CD16^+$), and non-classical monocytes (3, $CD45^+$ $CD11b^+$ CX_3CR1^+ $CD14^-$ $CD16^{++}$).

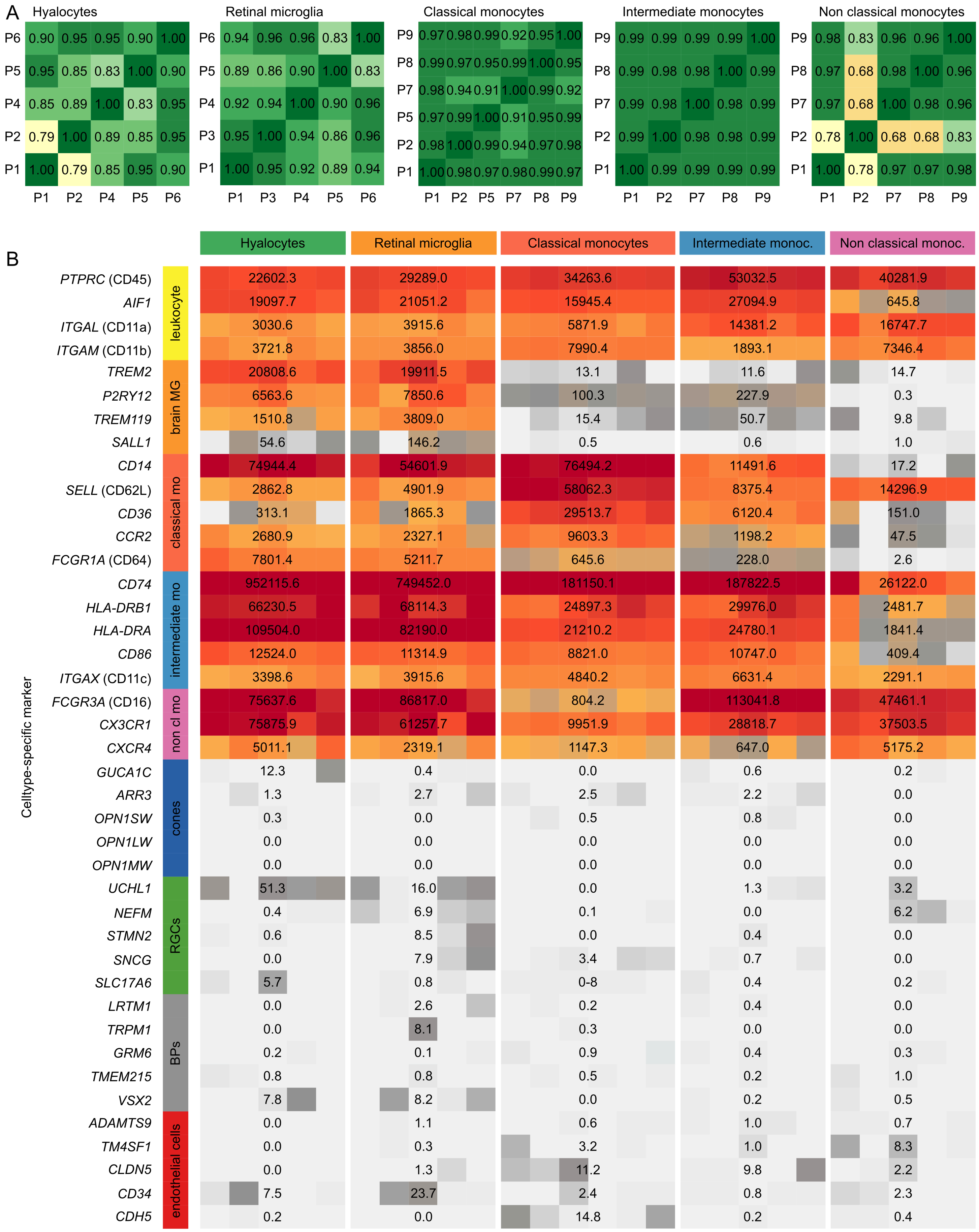

Supplementary figure 3: Quality and purity check. (A): Correlation plots visualizing Pearson correlation coefficients between any two patients (P) for all cell populations. (B): Expression of known celltype-specific marker genes in all 5 immune cell populations. Each column represents one cell population and each row one marker gene. Groups of marker genes are visualized for each celltype in the row annotation on the left. Numbers correspond to mean of normalized reads per group. Abbreviations: mo: monocyte, non cl: non classical, MG: microglia, RGC: retinal ganglion cell, BP: bipolar cell.

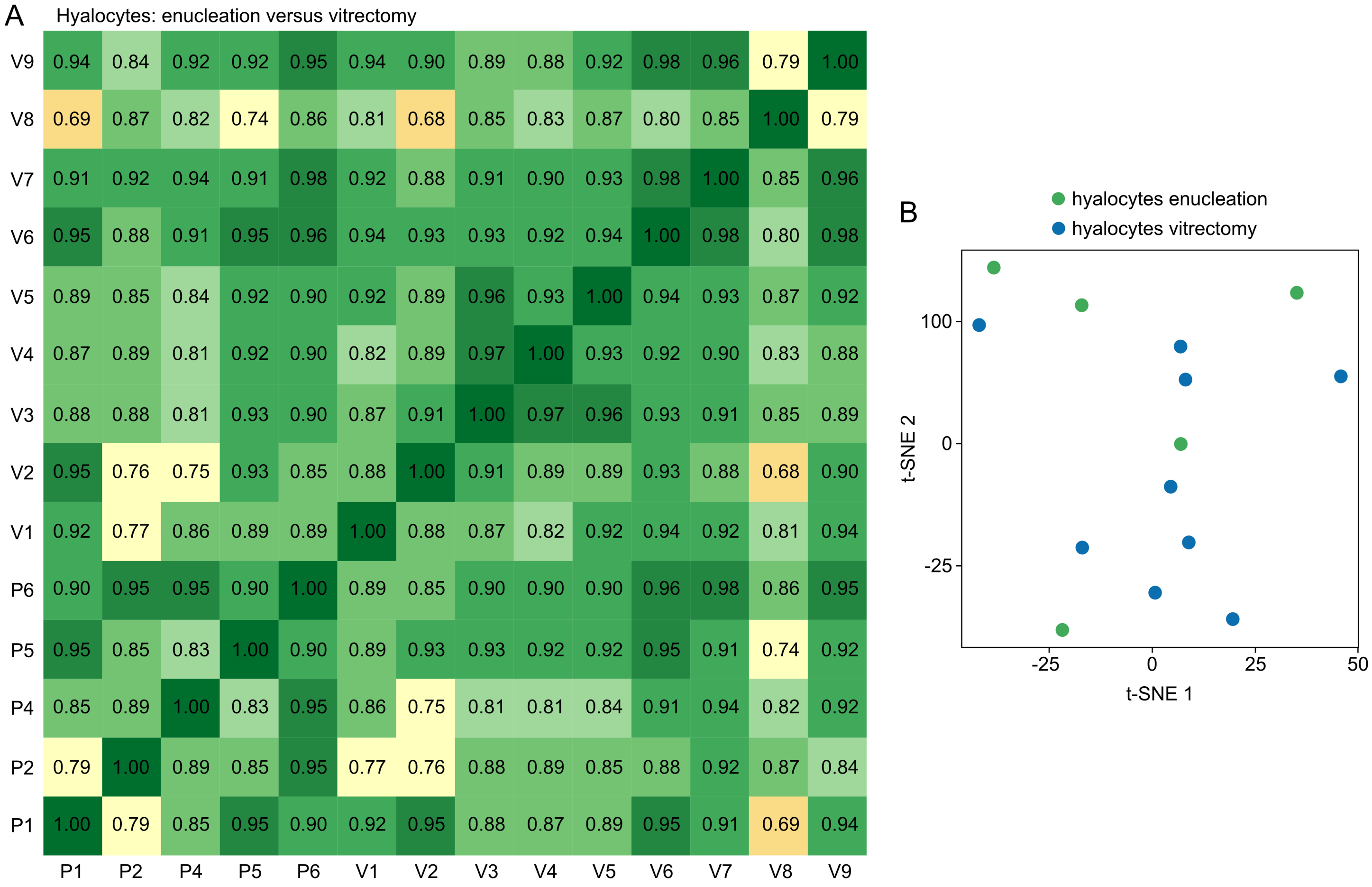

Supplementary figure 4: Comparison of transcriptional profiles of hyalocytes isolated from enucleated melanoma eyes (P1-6) and after vitrectomy (macular pucker, macular hole) (V1-9). (A): Correlation plots visualizing Pearson correlation coefficients between any two patients for hyalocytes from both groups. (B): Unsupervised cluster analysis using t-Distributed Stochastic Neighbor Embedding (t-SNE) of hyalocytes from melanoma eyes (enucleation) and after vitrectomy reveals no significant differences between the two groups.

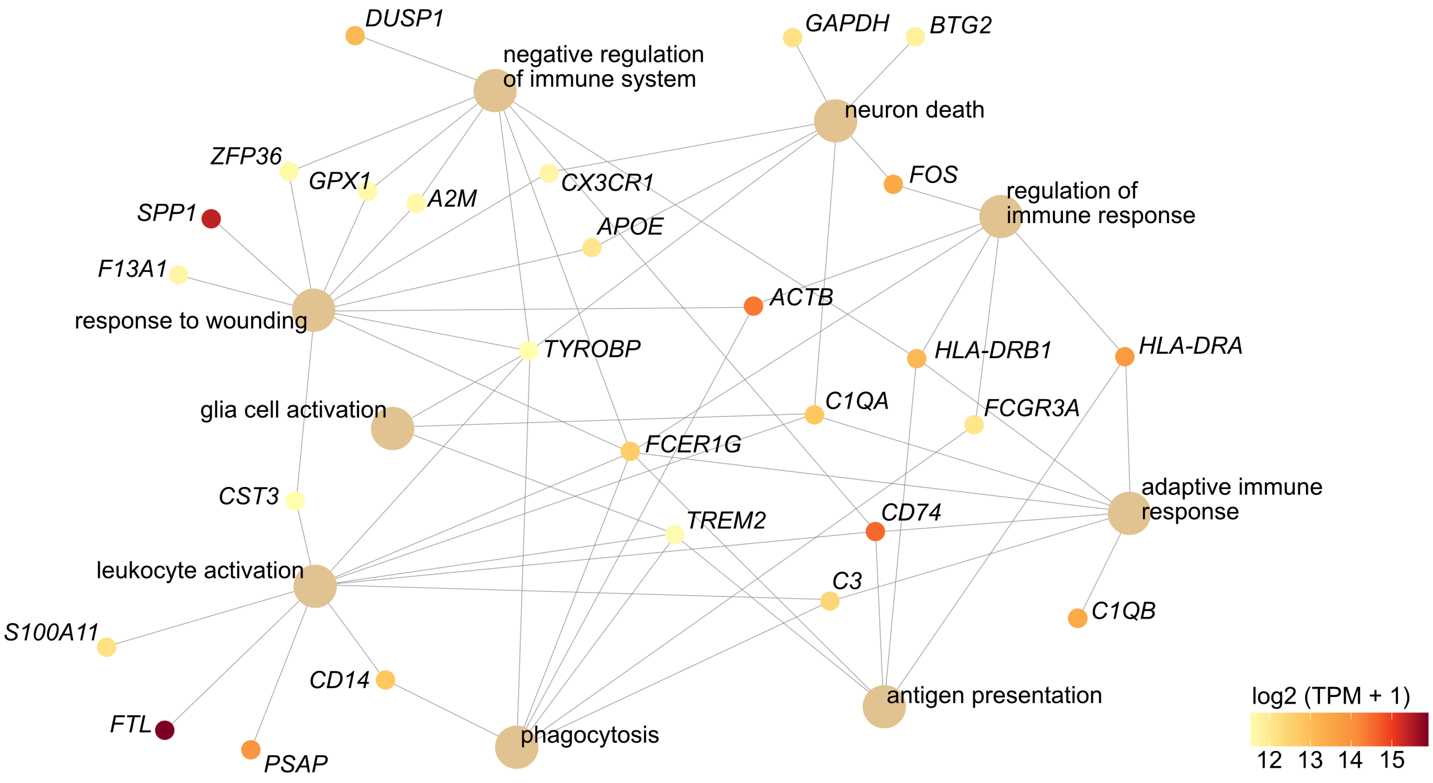

Supplementary figure 5: Network diagram illustrating the genes associated with the most significantly enriched GO biological processes in hyalocytes from Figure 1C, with the color representing the expression of each gene. TPM: transcripts per million.

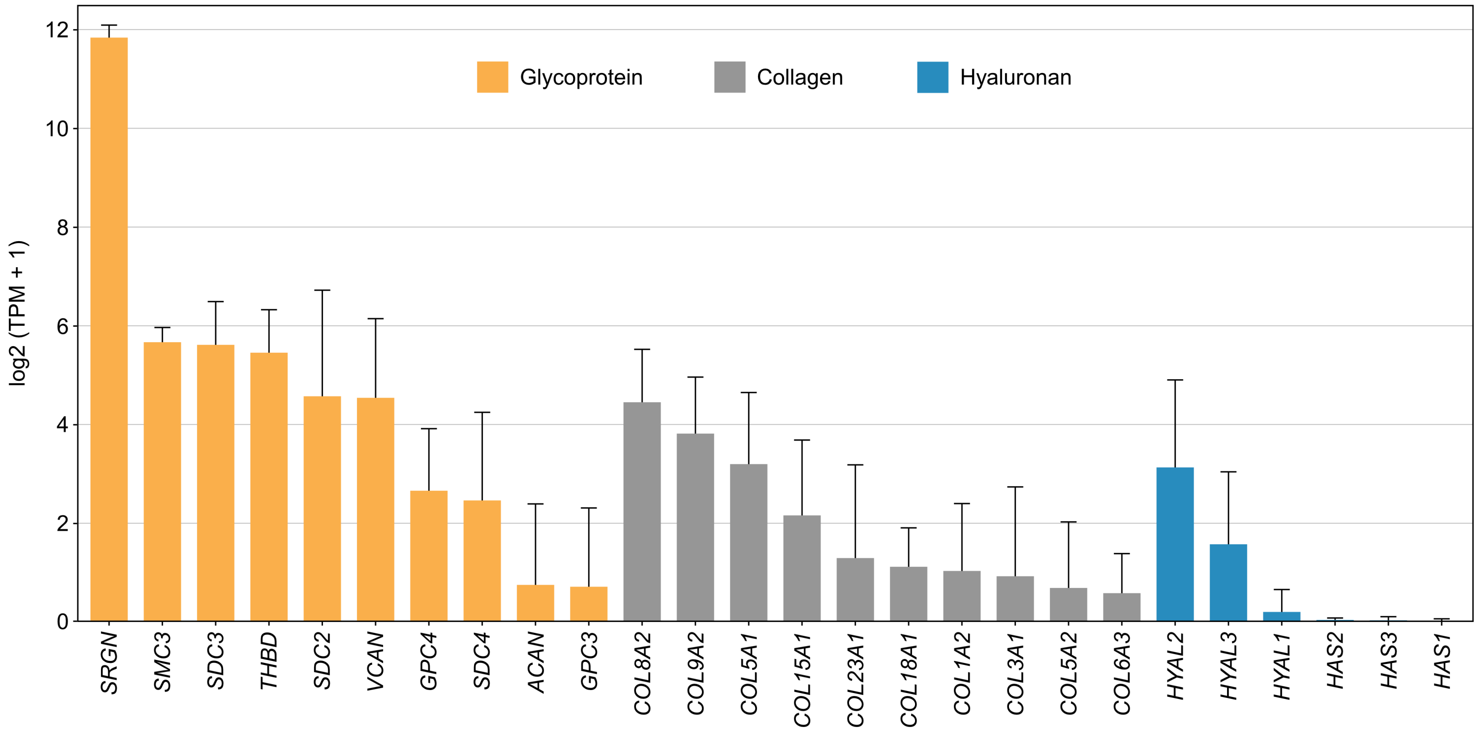

Supplementary figure 6: Expression of genes encoding for glycoproteins (yellow), collagen (grey) and hyaluronan (blue) in human hyalocytes. In each category, the ten highest expressed genes are visualized. Bar's height: mean expression, error bar: standard deviation. TPM: transcripts per million.
